# Editorial: Immunomics in aquaculture: deciphering the immune landscape of aquacultured animals through omics technologies

**DOI:** 10.3389/fimmu.2026.1788094

**Published:** 2026-02-09

**Authors:** Jorge Galindo-Villegas, Patricia Pereiro

**Affiliations:** 1 Department of Genomics, Faculty of Biosciences and Aquaculture, Nord University, Bodø, Norway; 2 Institute of Marine Research (IIM), Spanish National Research Council (CSIC), Vigo, Spain

**Keywords:** disease resistance, functional genomics, host-pathogen interactions, immunometabolism, molecular phenotyping, single-cell transcriptomics, systems Immunology, precision aquaculture

## From descriptive immunology to systems-level understanding

Advances in high-throughput omics technologies have reshaped modern biological research, with aquaculture standing among the disciplines most profoundly transformed. Genomics, epigenomics, metagenomics, transcriptomics, proteomics, and metabolomics now provide unprecedented resolution of the molecular and cellular processes that govern health, performance, and resilience in aquacultured species. This technological convergence is driving a transition toward precision aquaculture, in which data-driven insights inform selective breeding, nutritional strategies, and evidence-based disease management. Within this framework, immunomics has emerged as a unifying concept that integrates multiple molecular layers to interrogate immune function as a dynamic system. Classical descriptive immunology in aquaculture has largely relied on limited panels of generic immune markers, bulk tissue analyses, and static end-point measurements, approaches that provide only partial insight into the complexity of host-pathogen interactions. In contrast, immunomics enables the simultaneous interrogation of genetic architecture, cellular heterogeneity, transcriptional programs, and metabolic state, thereby transforming immune responses from qualitative descriptors into quantitative, mechanistically informed, and increasingly predictive biological systems. Metagenomics further expands this perspective by incorporating host-associated microbial communities as integral components of immune development and disease susceptibility.

## From genomic architecture to functional disease resistance

Genomics provides the foundational blueprint for immune potential and disease resistance in aquaculture species. Whole-genome sequencing (WGS) and resequencing approaches have enabled the identification of single nucleotide polymorphisms (SNPs) and structural variants associated with resistance phenotypes in fish (1), mollusks (2), and crustaceans (3). These studies have demonstrated that resistance traits are often oligogenic and shaped by complex genetic architectures rather than single major-effect loci (4). Importantly, the power of genomics is amplified when coupled with functional layers of analysis. Zhu et al. exemplify this integrative approach by combining genome-wide association studies (GWAS) with transcriptomic profiling to dissect resistance of *Larimichthys polyactis* to *Pseudomonas plecoglossicida*. Their identification of resistance-associated loci and the cGAS-STING signaling pathway, with *sting1* emerging as a key candidate gene, illustrates how genomic signals can be mechanistically anchored to immune pathways of direct biological relevance. Similarly, Pereiro et al. demonstrate that genetic resistance may be partially constitutive. By comparing resistant and susceptible full-sibling families of turbot (*Scophthalmus maximus*), they revealed profound baseline transcriptomic differences in immune organs even in the absence of infection. Upon challenge with *Aeromonas salmonicida* subsp. *salmonicida* resistant fish displayed more controlled inflammatory responses and enhanced antigen presentation, reinforcing the concept that disease resistance is encoded not only in inducible responses but also in preconfigured immune states.

## Transcriptomics as the cornerstone of aquaculture immunomics

Transcriptomics remains the most widely applied immunomics approach in aquaculture, owing to its affordability, sensitivity, and mature analytical pipelines. Bulk RNA sequencing has been instrumental in identifying immune pathways activated during bacterial (5), viral (6), and parasitic (7) infections, and in linking transcriptional programs to phenotypic outcomes such as survival, pathology, and tolerance. Hao et al. provide a clear example of the power of temporal transcriptomics by profiling the hepatopancreas of *Macrobrachium rosenbergii* following infection with Decapod iridescent virus 1 (DIV1). Their analysis revealed coordinated activation of lysosomal, phagosomal, and C-type lectin receptor pathways, alongside pronounced metabolic remodeling. These findings highlight how time-resolved transcriptomics can capture the dynamic progression of host defense mechanisms and identify intervention-relevant pathways. In parallel, transcriptomic studies have expanded beyond protein-coding genes to encompass non-coding regulatory layers. Long non-coding RNAs (lncRNAs) (8), circular RNAs (circRNAs) (9), and small non-coding RNAs (sncRNAs) (10) are now recognized as integral components of immune regulation, contributing to transcriptional control across diverse aquaculture species.

## Resolving cellular heterogeneity through single-cell immunomics

While bulk transcriptomics has transformed the field, it inherently averages signals across heterogeneous cell populations. Single-cell and single-nucleus RNA sequencing (scRNA-Seq and snRNA-Seq) now enable the resolution of immune cell diversity, lineage specialization, and cell-type-specific response trajectories (11). Aldersey et al. illustrate the transformative potential of single-cell immunomics through the analysis of the hepatopancreas of Pacific white shrimp (*Litopenaeus vannamei*) during *Vibrio parahaemolyticus* infection. By resolving distinct cellular populations, the study uncovered cell-specific activation of pathogen recognition receptors, humoral effectors, and potential toxin-responsive pathways, alongside profound metabolic reprogramming. These findings provide high-resolution insight into shrimp host-pathogen interactions and underscore the importance of cellular context at the single-cell resolution when interpreting immune responses. Despite these advances, single-cell immunomics in aquaculture species remains constrained by the absence, incompleteness, or fragmentation of references genomes, limited annotation of immune cell markers, and challenges in cross-species cell-type inference. Continued efforts in genome improvement, immune cell atlas generation, and comparative immunology will be essential to fully exploit scRNA-Seq and snRNA-Seq technologies in non-model aquatic organisms.

## Immunometabolism as a central axis of host defense

Beyond gene expression, immune competence is tightly coupled to metabolic state. Proteomics and metabolomics offer direct openings into the effector molecules and biochemical pathways that execute and sustain immune responses. Although proteomic approaches face challenges related to cost and data complexity (12), they have yielded valuable insights into conserved immune mechanisms across vertebrates (13, 14) and invertebrates (15). Metabolomics has revealed that immune activation is intimately linked to metabolic remodeling. By capturing changes in small-molecule metabolites, metabolomic analyses illuminate how immune activation reshapes energy allocation and redox balance (16). This immunometabolic perspective is strongly reinforced across studies in this Research Topic. Both Hao et al. and Aldersey et al. report extensive metabolic reprogramming accompanying immune activation, while Zhou et al. demonstrate how oxidative stress markers and antioxidant enzyme activities reflect immune perturbations during ectoparasitic infection. For instance, shifts toward glycolytic metabolism are associated with pro-inflammatory effector functions, including enhanced phagocytosis and antimicrobial activity, whereas oxidative and lipid-based metabolic programs support immune resolution, antioxidant defense, and tissue repair (6, 13, 14). Such metabolic polarization is evident in both vertebrate and invertebrate aquaculture species, underscoring immunometabolism as a functional driver. Together, these studies position immunometabolism not as a secondary consequence, but as a core axis of host-pathogen interaction in aquatic cultured species.

## Barrier tissues and peripheral immune landscapes

Traditionally, immunological studies in fish have focused on primary immune organs such as the head kidney and spleen. However, omics technologies are increasingly revealing the importance of barrier tissues and peripheral organs as active immune interfaces. Zhou et al. investigated the response of *Larimichthys crocea* to infection with the scuticociliate parasite *Metanophrys* sp. by integrating enzymatic immune profiling across multiple tissues with skin transcriptomics. Their results identify the skin as a dynamic immune barrier, where antioxidant defenses, osmoregulatory (Na^+^/K^+^-ATPase) enzymes and immune effectors are coordinately regulated in response to the ectoparasite infestation. Comparison of the skin transcriptome between control and infested fish revealed numerous differentially expressed genes, providing valuable information about mechanisms of defense against this pathogen. Extending this tissue-centric view, Appel et al. analyzed brain transcriptomes of Nile tilapia (*Oreochromis niloticus*) infected with distinct *Streptococcus agalactiae* serotypes. The contrasting immune and neuroendocrine responses elicited by different serotypes emphasize the functional relevance of non-canonical immune tissues. Importantly, omics-based analyses increasingly point to bidirectional crosstalk between peripheral barrier tissues and systemic immune organs, including emerging evidence for gut-brain communication and liver-centered immunometabolic axes (17). Signals originating in the skin, gut, brain, or liver can reshape immune cell composition, metabolic state, and activation programs in central organs such as the head kidney and spleen, reinforcing immunity as a distributed, interconnected system rather than a collection of isolated compartments.

## Pathogen diversity and the need for context-aware immunomics

A recurring theme across studies in this Research Topic is that immune responses are profoundly shaped by pathogen identity, strain, and virulence strategy. The serotype-specific responses reported by Appel et al. underscore the risk of oversimplification when extrapolating immune mechanisms across pathogens. These findings highlight the necessity of context-aware immunomics approaches that explicitly account for pathogen diversity when designing vaccines, immunostimulants, and experimental infection models.

## Concluding perspectives

**Figure 1:**
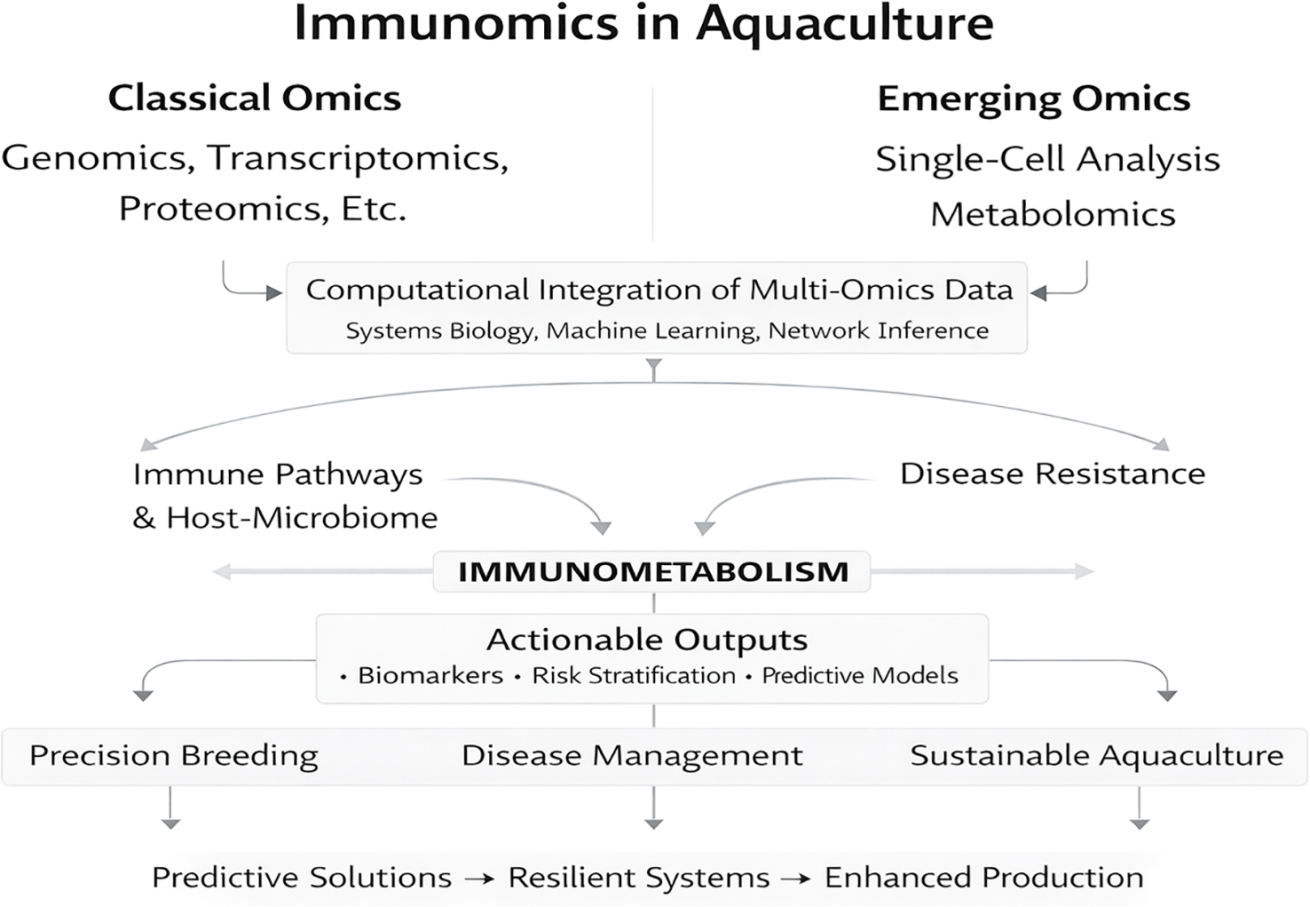
Integrated immunomics framework in aquaculture. Classical and emerging omics layers (including genomics, transcriptomics, proteomics, metabolomics, metagenomics, and single-cell technologies) are computationally integrated to illustrate the scope of immunomics approaches capable of resolving immune complexity across biological scales and informing biomarker discovery, predictive modeling, and translational aquaculture strategies.

Collectively, the studies assembled in this Research Topic illustrate the transformative capacity of immunomics to resolve the complexity of immune systems in aquaculture species (**Figure 1**). By integrating classical and emerging omics layers, now increasingly combined with single-cell and systems-level resolution, immunomics is driving a conceptual shift from descriptive immunology toward predictive, mechanistically grounded strategies. As omics platforms continue to mature and converge, immunomics will play a central role in shaping sustainable, resilient, and precision-driven aquaculture systems. Beyond improving disease management, these approaches provide a foundation for rational breeding, targeted nutritional interventions, and anticipatory health strategies capable of supporting the growing global demand for aquatic food production.
